# Diaphragmatic ultrasound and its relationship to breathing effort and load: a prospective observational study

**DOI:** 10.1186/s13054-025-05436-1

**Published:** 2025-05-13

**Authors:** Apostolos–Alkiviadis Menis, Vasiliki Tsolaki, Maria–Eirini Papadonta, Vasileios Vazgiourakis, Konstantinos Mantzarlis, Epaminondas Zakynthinos, Demosthenes Makris

**Affiliations:** 1 Intensive Care Unit, University Hospital of Larissa, Larissa, Greece; 2 Medical School, University of Thessaly, Larissa, Greece

**Keywords:** Ultrasound, Diaphragm, Load, Esophageal pressure, Transdiaphragmatic pressure

## Abstract

**Background:**

Failure to wean from invasive mechanical ventilation is multifactorial, with diaphragmatic dysfunction a significant contributing factor. Diaphragmatic function can be easily and non-invasively assessed by ultrasound. However, it remains unknown how ultrasound measurements of diaphragm function are affected by changes in apparent work of breathing.

**Methods:**

In patients undergoing weaning from mechanical ventilation, we evaluated diaphragmatic ultrasound measurements [diaphragmatic excursion (Dex), diaphragmatic thickening fraction (Tfdi)] simultaneously with manometric indices of breathing effort and load [esophageal pressure swings (ΔPes), transdiaphragmatic pressure swings (ΔPdi), and the pressure–time product of esophageal pressure (PTPes)]. These assessments were performed during two distinct phases; during an unassisted spontaneous breathing trial (phase SBT) and during an inspiratory resistive loading with 30 cmH_2_O/L/s (phase IRL), applied during the same SBT. Our primary aim was to evaluate the relationship between diaphragmatic ultrasound and breathing effort using the method of repeated measures correlation.

**Results:**

Forty-nine patients were enrolled. Dex correlated with ΔPes (r = 0.5, *p* < 0.001), ΔPdi (r = 0.55, *p* =  < 0.001) and PTPes (r = 0.32, *p* = 0.031). Tfdi did not correlate with ΔPes (r = 0.27, *p* = 0.052), ΔPdi (r = 0.2, *p* = 0.235) and PTPes (r = 0.24, *p* = 0.110). Dex and Tfdi increased during IRL compared to SBT [1.44(0.89–1.96) vs. 1.05(0.7–1.59), *p* = 0.002], [0.55(± 0.32) vs 0.46(± 0.2), *p* = 0.019] as did Pes, Pdi and PTPes [(11.87 (7.86, 18.32) vs. 6.8 (4.6–10.23), *p* < 0.001), (10.89 (± 6.42) vs. 7.94 (± 3.81), *p* < 0.001), and (181.10 (108.34, 311.7) vs. 97.52 (55.96–179.87), *p* < 0.001), respectively].

**Conclusion:**

In critical care patients spontaneously breathing under resistive load, diaphragmatic excursion had a weak to moderate correlation with indices of breathing effort and differed between weaning success and failure.

## Introduction

Failure to wean from invasive mechanical ventilation is a multifactorial issue, with diaphragmatic dysfunction potentially being a significant factor [1, 2]. During spontaneous breathing trials, patients with diaphragmatic dysfunction may struggle with the increased breathing load relative to having been on full ventilator support, leading to weaning failure [2]. An easy-to-use bedside tool that can reliably identify diaphragmatic dysfunction exacerbated by an increased work of breathing could therefore be useful in identifying patients at high risk of weaning failure.

Esophageal (ΔPes) or transdiaphragmatic pressure swings (ΔPdi) and the pressure–time product of the esophageal pressure (PTPes) are indices known to reflect breathing effort and present an easier alternative for its assessment compared to bilateral twitch transdiaphragmatic pressure measurements, which is the gold standard [3]. However, all these methods present significant challenges as they are relatively invasive, can be time-consuming and require specialized equipment [3].

In contrast, diaphragmatic ultrasound is an easy to use, non-invasive tool for diaphragmatic function assessment [4–6]. The two main indices evaluated with diaphragmatic ultrasound are diaphragmatic excursion (Dex), the caudal inspiratory movement of the diaphragm, and diaphragmatic thickening fraction (Tfdi), the percentage of inspiratory thickening of the diaphragm [5, 6]. Previous studies have assesed its efficacy in predicting weaning outcomes [7–9] and estimating breathing effort [10–20]. However, specifically in relation to breathing effort, the results have been ambiguous and most studies have focused mainly on patients under positive pressure ventilation [13–21], a modality that alters diaphragmatic mechanics and can potentially confound Dex and Tfdi measurements [5, 17, 22].

In this prospective observational study, we assessed patients undergoing a spontaneous breathing trial with a T-piece while applying an external inspiratory resistive load, mimicking common conditions in critical care patients [23, 24]. We hypothesized that diaphragmatic ultrasound indices (Dex and Tfdi) would correlate with invasively measured breathing effort parameters (ΔPes, ΔPdi, and PTPes) under conditions of increased inspiratory load.

## Materials and methods

### Setting and participants

This prospective observational study was conducted at a tertiary academic intensive care unit (ICU) in Larissa, Greece. Recruitment of the subjects and data collection was conducted during a 1-year period whereas data analysis was completed within 2 months. Subjects were recruited by consecutive sampling if they fulfilled criteria indicating readiness for weaning [25]. Subjects < 18 years old, pregnant women and subjects with active esophageal/gastric/diaphragmatic disease were excluded from the study.

The study was approved by the Ethics Committee of the University Hospital of Larissa. The procedures followed were in accordance with the ethical standards of the Ethics Committees of the University Hospital of Larissa and with the Helsinki Declaration of 1975. Informed written consent was obtained from the subjects (or next of kin) prior to enrollment. The trial was registered on clinicaltrial.gov (NCT03802383).

### Study design and outcomes

Subjects deemed ready for weaning by the treating physician underwent a thirty-minute spontaneous breathing trial (SBT) using a T-piece [25]. During the SBT, following a five-minute period to ensure clinical stability, measurements of ΔPes and Pgas were conducted. Simultaneous diaphragmatic ultrasound was also performed to assess Dex and Tfdi (phase SBT). Immediately after these measurements were completed, a one-minute IRL was conducted during the same SBT, with the same parameters being measured (phase IRL). All the measurements were completed between the 5th and 10th minute of the SBT. For each phase, the analysis included the mean values from ten consecutive breaths. Artifacts caused by coughing or movement and any signal interference (i.e. chest wall motion or lung interference) altering the interpretability of the data were excluded and we used the next available breath without artifacts. All the measurements were performed with the patients in the 30–45 degrees position. The synchronization of ultrasound measurements with esophageal manometry was achieved by aligning the ultrasound machine and the personal computer running the dedicated software to operate on the same time settings.

The primary aim of this study was to assess the relationship between Dex and Tfdi with ΔPes, ΔPdi and PTPes. Secondarily, we assessed weaning outcome and its relationship to diaphragmatic ultrasound.

### Definitions

Weaning failure was defined either as a SBT failure or need for reintubation at 48 h; this was judged by the treating physician according to the local protocol, which was based on published criteria [25]. These criteria allowed for adequate categorization of all enrolled patients and are widely used in studies evaluating diaphragmatic ultrasound in weaning outcome prediction [8, 9], ensuring comparability and consistency among studies.

Readiness for weaning was assessed by the treating physician based on previously published criteria [25]: resolution of disease, adequate cough, absence of excessive tracheobronchial secretion, stable cardiovascular (minimal vasopressors, heart rate < 140 bpm, SBP 90–160 mmHg) and pulmonary function (no significant respiratory acidosis, respiratory rate < 35 breaths/min), adequate oxygenation (SaO_2_ > 90% on FiO_2_ < 40%, PEEP < 8 cmH2O) and mentation.

Failure of the SBT was defined by: a) objective criteria of failure such as PaO_2_ < 50–60 mmHg on FiO_2_ > 0.5 or SaO_2_ < 90%, PaCO_2_ > 50 mmHg or an increase in PaCO_2_ > 8 mmHg, respiratory rate > 35 breaths/min, heart rate > 140 beats, Systolic BP > 180 mmHg or Systolic BP < 90 mmHg, cardiac arrhythmias, and b) subjective criteria such as agitation and anxiety, depressed mental status, diaphoresis, cyanosis, evidence of increasing effort, increased accessory muscle activity, facial signs of distress and dyspnea [25].

### Diaphragmatic ultrasound and esophageal pressure

Airway pressures, tidal volume (Vt) and flow were continuously recorded using a heated pneumotachometer (Series 3813; Hans Rudolph Inc. USA) and a Research Pneumotach System (RSS100-HR; Hans Rudolph Inc. USA). ΔPes and gastric pressures (Pgas) were measured as described in the literature [26, 27]. Both balloons were connected to a pressure transducer (RSS-100HR, Hans Rudolph Inc. USA) and their signal was graphically depicted in a personal computer through dedicated software (RSS 100-HR for Windows v. 3.07.08, sample rate 50 Hz). The signal extraction and analysis were conducted using an image processing and analysis software (ImageJ 1.53 k, NIH, United States).

ΔPdi was calculated as Pes—Pgas [28]. ΔPes and ΔPdi were calculated as the absolute difference between the end expiratory and peak inspiratory value of Pes and Pdi, respectively. PTPes was calculated as previously described in the literature [29]. The chest wall compliance was estimated as 4% of predicted vital capacity, a theoretical value that is commonly used in existing literature. [30]. The inspiratory (Ti), expiratory (Texp) and total respiratory time (Ttot) were electronically calculated from the dedicated software (RSS 100-HR for Windows v. 3.07.08). Mean inspiratory flow (MIF) was calculated as Vt/Ti [31]. Rapid shallow breathing index was calculated as Vt/RR [32].

A modified T-tube setup was used to apply inspiratory resistive loading (IRL). In this context, "limb" refers to the modified inspiratory and expiratory pathways within the T-tube setup. The central “limb” of the T-tube side was connected to the artificial airway, the T-tube part that was connected to the oxygen supply was fitted with a resistive load (30 cm H₂O/L/s), while the other part was equipped with a one-way valve that allowed only expiratory flow. Expiration was not occluded, but it was directed through the one-way valve, ensuring unidirectional airflow. IRL was applied for one minute, and patients were instructed to notify researchers if they experienced discomfort to allow for immediate termination of the maneuver.

All ultrasound measurements were performed on the right hemidiaphragm, due to the limited acoustic window of the left, using an ultrasound (System Vivid™ E95, GE Medical Systems, USA-Philips iE33, Philips Medical, USA) equipped with a linear 3–11 MHz and a phased array 3.5–5 MHz probe. Dex was measured using a phased-array probe placed in the right subcostal region with a medial, cephalad and dorsal orientation, using the liver as acoustic window, with the diaphragm being depicted as a hyperechoic structure moving caudally during the inspiration. Tfdi [(peak-inspiratory thickness – end expiratory thickness)/ end expiratory thickness)] was measured using the linear probe placed perpendicularly on the chest wall, on the right midaxillary line (8th-11th rib) [5]. In each patient the probe location was adjusted so as to acquire the best visualization possible. The right diaphragm was identified as a three-layered structure (two hyperechoic lines with a middle hypoechoic line). Each ultrasound study was performed by one researcher (V.T. or V.V.). The measurements were then analyzed at a later time by both researchers and any disagreement in calculation was judged by a third part (D.M.). The manometric measurements were performed by AAM and MEP. AAM and MEP were blinded to the results of the ultrasound while VT and VV were blinded to the results of the manometry. All the aforementioned researchers were blinded to weaning related decisions. We assessed intra- and interobserver variability for absolute agreement between measurements per datapoint, for each ultrasound variable (Dex, Tfdi), using intraclass coefficient separately for each dataset (SBT and IRL).

### Statistical analysis

Results are reported as mean (± standard deviation) if normally distributed or as median (IQR) otherwise. Outliers were not excluded. Assumption of normality was tested using the Shapiro–Wilk test. A Student’s paired t-test was used for normally distributed data and a Wilcoxon’s rank test for non-parametrical data. Repeated measures correlation (95% CI) was computed to determine the within-patient relationship between diaphragmatic ultrasound and breathing effort. This method takes into consideration the interindividual variability and the independence of repeated measures between individuals [33]. A *p* value < 0.05 was considered significant. Statistical analysis was conducted using Jamovi version 1.6.23. The repeated measures correlation was performed using the ‘rmcorr’ R package [R version 4.3.2 (RStudio 2023.12.0 Build 369)]. The measurements were compared phase-by-phase and not breath-by breath, using the average of ten consecutive breaths for each variable. As to our knowledge there was no previous similar study in critical care patients, we used a convenience sample based on previous physiological studies.

## Results

Fifty-seven patients were assessed for eligibility. Forty-nine patients were enrolled. Twenty-six succeeded in weaning. In the failure group, fifteen patients failed the SBT. The APACHE-II [34, 35] score in this cohort was 16.21 (± 7.18). Basic demographic characteristics are depicted on Table 1. The ICC for intra and interobserver variability in SBT and IRL were 0.98 (0.96, 0.97) and 0.95 (0.94, 0.96) and 0.97 (0.96, 0.98) and 0.93 (0.92, 0.94) respectively. In three patients the IRL phase lasted approximately two minutes, the reason being technical difficulties in acquiring adequate ultrasound images. Gastric pressure was measured in thirty-six patients (not accepting a second (gastric) balloon, n = 6, continuous artifacts during the timeframe of recordings, n = 3 stomach discomfort, n = 4).

**Table 1:** Basic demographic characteristics

	Total (N = 49)	Weaning Success (N = 26)	Weaning failure (N = 23)	*p*-value
Age, years	60.85 (± 16.11)	66.5 (51.25–75.5)	62.5 (49.75–73.25)	0.694
Sex, male	42 (85.7%)	23 (88%)	19 (82%)	0.5592
Days of MV	14 (8, 20)	12.5 (5.25–17.75)	15 (9.5–23.5)	0.306
APACHE-II	16.21 (± 7.18)	15 (9–21)	16 (13.25–22.75)	0.436
*Reason of intubation*				0.953
Respiratory failure	15 (31%)	8 (31%)	7 (30%)	
Traumatic brain injury	10 (20%)	6 (23%)	4 (17%)	
Neurological	11 (22.4%)	5 (19%)	6 (26%)	
Post-operative	8 (16.3%)	5 (19%)	3 (12%)	
Septic shock	3 (33.3%)	1 (4%)	2 (9%)	
Cardiac arrest	2 (4%)	1 (4%)	1 (4%)	

MV, mechanical ventilation; APACHE II, Acute Physiology and Chonic Health Evaluation II

### Relationship between ultrasound and manometric indices

**Fig. 1:**
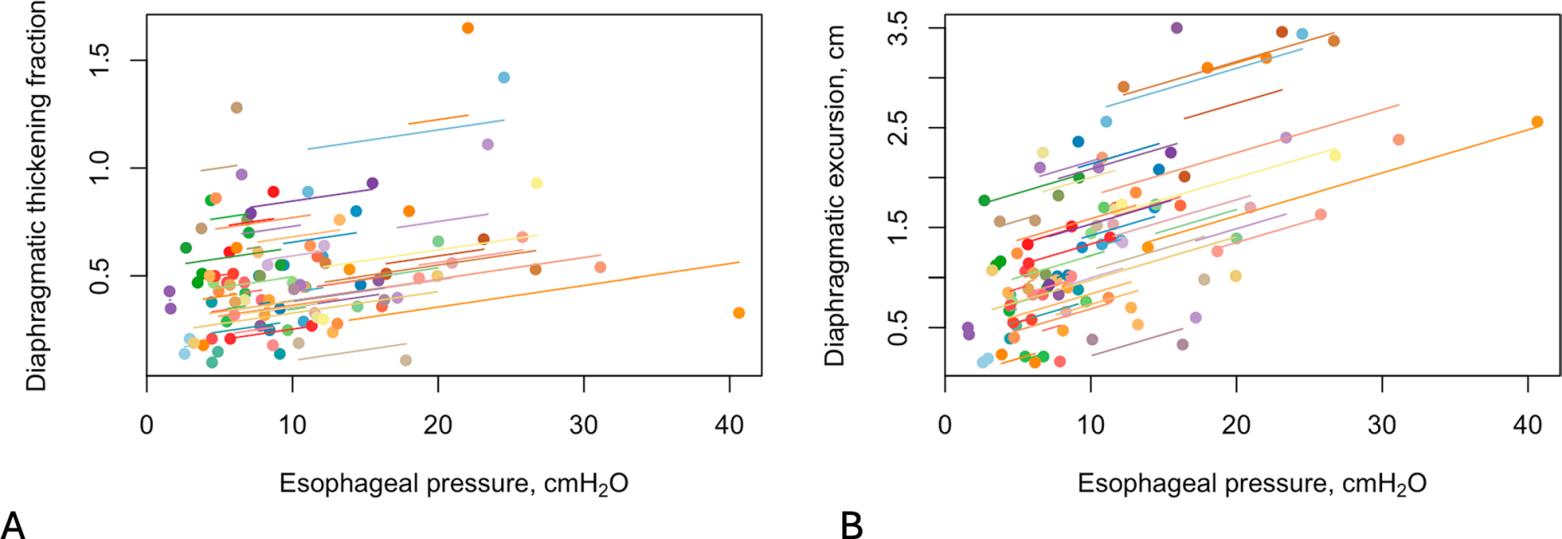
**A** The relationship of diaphragmatic thickening fraction with esophageal pressure, and **B** the relationship of diaphragmatic excursion with esophageal pressure. Observations from the same participant are given the same color, with corresponding lines to show the best fit for each participant

**Fig. 2:**
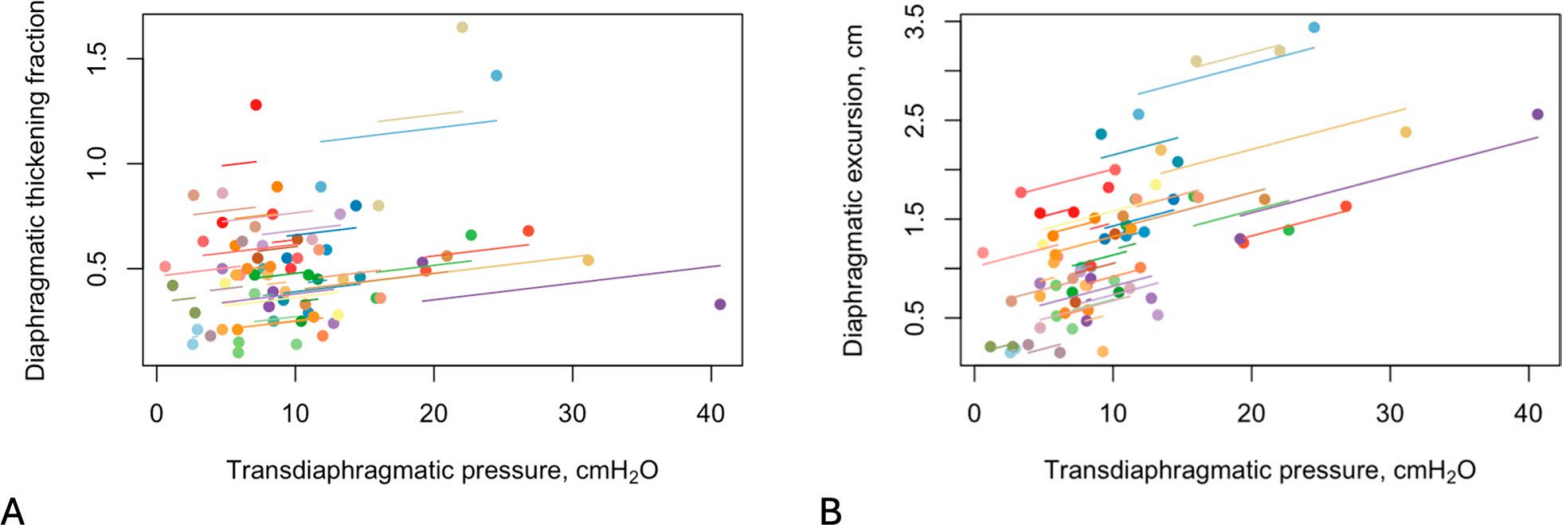
**A** The relationship of diaphragmatic thickening fraction with transdiaphragmatic pressure, and **B** the relationship of diaphragmatic excursion with transdiaphragmatic pressure. Observations from the same participant are given the same color, with corresponding lines to show the best fit for each participant

Tfdi did not have any correlation with ΔPes (r = 0.27, *p* = 0.052), ΔPdi (r = 0.2, *p* = 0.235) (Fig. 1A and 2A) and PTPes (r = 0.24, *p* = 0.110). Dex correlated with ΔPes (r = 0.5, *p* < 0.001), ΔPdi (r = 0.55, *p* =  < 0.001) (Fig. 1B, 2B) and PTPes (r = 0.32, *p* = 0.031).

### Comparison between weaning outcomes

**Fig. 3:**
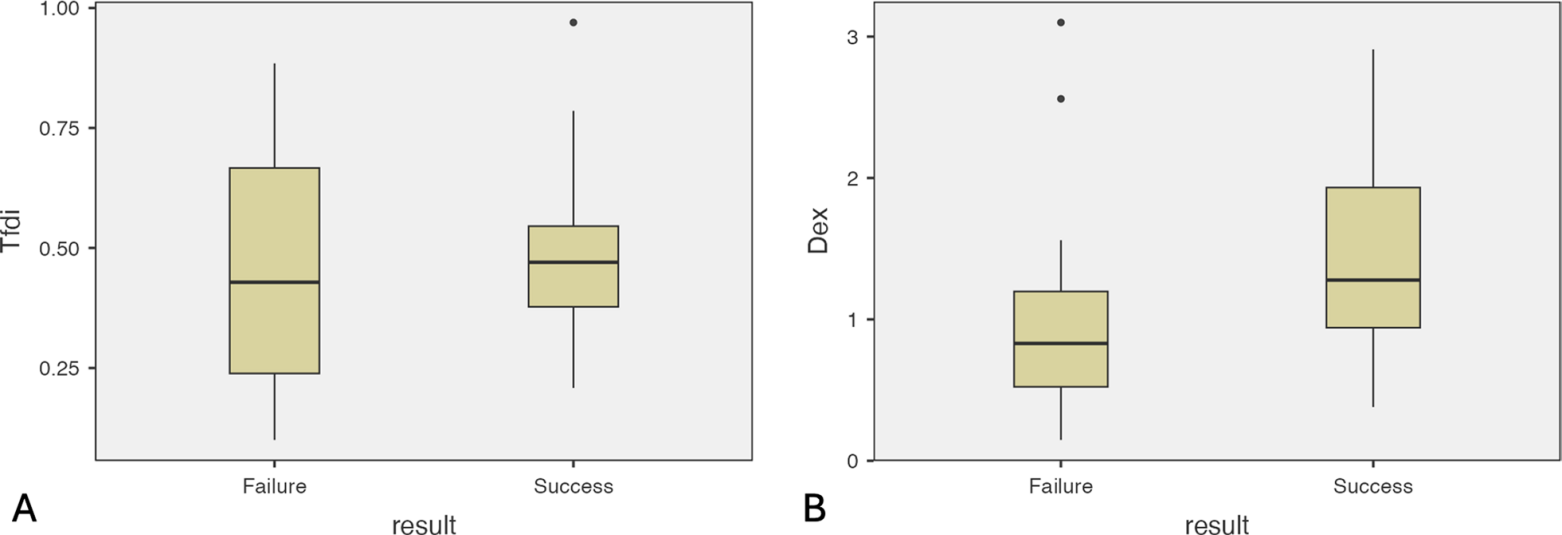
**A** Diaphragmatic thickening fraction during spontaneous breathing trial in patients succeeding weaning and in patients failing weaning. **B** Diaphragmatic excursion during spontaneous breathing trial in patients succeeding weaning and in patients failing weaning

Tfdi was similar between weaning success and failure both in tidal and resistive breathing (*p* = 0.336) and (*p* = 0.545), respectively (Fig. 3A). Patients who succeeded in weaning had higher Dex both in tidal and resistive breathing [1.28 (0.94–1.93) cm vs 0.83 (0.51–1.26) cm, p = 0.02], [1.7 (1.36–2.19) cm vs 1.01 (0.58–1.51) cm, p = 0.017] respectively (Fig. 3B). (Table 2).

**Table 2:** Comparison of diaphragmatic ultrasound between weaning outcomes

	Weaning success	Weaning failure	*p*-value
Dex	1.28 (0.94–1.93)	0.83 (0.51–1.26)	0.02
Dex_res_	1.7 (1.36–2.19)	1.01 (0.58–1.51)	0.017
Tfdi	0.47 (0.38–0.55)	0.43 (0.24–0.67)	0.336
Tfdi_res_	0.52 (0.36–0.67)	0.48 (0.35–0.62)	0.545
Vt	449.52 (± 163.87)	245.05 (± 119.12)	< 0.001
Vt_res_	240.68 (178.51–328.37)	188.31 (119.3–260.95)	0.045
RSBI	57.41 (39.38–77.59)	118.39 (99.1–173.13)	< 0.001
RSBI_res_	88.96 (75.4–160.78)	201.27 (130.56–257.84)	0.002

Dex = diaphragmatic excursion, Dex,res = diaphragmatic excursion during resistive breathing, Tfdi = diaphragmatic thickening fraction, Tfdi,res = diaphragmatic thickening fraction during resistive breathing, Vt = Tidal Volume, Vt_res_ = tidal volume during resistive breathing, RSBI = rapid shallow breathing index, RSBI_res_ = rapid shallow breathing index during resistive breathing. Table entries represent means (± SD), medians (25th, 75th interquartile range. Comparisons between groups were performed with Student’s t test or Mann–Whitney U test respectively. P values represent the difference between spontaneous and resistive breathing

RSBI increased under IRL in both weaning success and failure groups (*p* < 0.001 and *p* = 0.016, respectively). Vt decreased under IRL (*p* < 0.001) in participants; the change was more prominent in weaning success group (*p* = 0.001) (see Table 2).

### Comparison between spontaneous and resistive breathing

During IRL Respiratory Rate (RR), Dex, Tfdi, ΔPes, ΔPdi, PTPes and Ti/Ttot increased whereas Vt, Texp and MIF decreased (Table 3). During spontaneous breathing Dex correlated with Vt (rho = 0.3, *p* = 0.037), while in IRL it did not (*p* = 0.226).

**Table 3:** Comparisons between spontaneous and resistive breathing

	Spontaneous breathing	Resistive breathing	p-value
*Basic respiratory parameters*			
RR, breaths/min	27 (± 9.29)	29.3 (± 9.15)	0.01
Vt, ml	354.08 (± 180.44)	243.41 (± 138.06)	< 0.001
RSBI, breaths/min/L	80.7 (47.16, 131.4)	146.8 (79.34, 201.9)	< 0.001
Ti, s	0.8 (0.7, 1)	0.8 (0.7, 1)	0.826
Texp, s	1.53 (1.04, 1.96)	1.14 (1.02, 1.44)	< 0.001
Ti/Ttot	0.36 (± 0.1)	0.41 (0.11)	0.005
MIF, ml/s	369.3 (284.68, 485.64)	265.2 (154.57, 343.67)	< 0.001
*Diaphragmatic ultrasound*			
Dex, cm	1.05 (0.7, 1.59)	1.44 (0.89, 1.96)	0.002
Tfdi	0.46 (± 0.2)	0.55 (± 0.32)	0.019
*Manometric indices*			
ΔPes, cmH_2_O	6.8 (4.6–10.23)	11.87 (7.86, 18.32)	< 0.001
ΔPdi, cmH_2_O	7.94 (± 3.81)	10.89 (± 6.42)	< 0.001
PTPes, cmH_2_O*s	97.52 (55.96–179.87)	181.10 (108.34, 311.7)	< 0.001

RR = respiratory rate, Vt = tidal volume, Ti = inspiratory time, Ti/Ttot = the ratio of inspiratory time to respiratory time, MIF = mean inspiratory flow, Dex = diaphragmatic excursion, Tfdi = diaphragmatic thickening fraction, ΔPes = esophageal pressure swings, ΔPdi = transdiaphragmatic pressure swings, PTPes = pressure–time product of the esophageal pressure. Table entries represent means (± SD), medians (25th, 75th interquartile range. Comparisons between groups were performed with Student’s t test or Mann–Whitney U test respectively. *P* values represent the difference between spontaneous and resistive breathing

## Discussion

This study aimed to evaluate Dex and Tfdi as non-invasive estimates of breathing effort under inspiratory resistive load. Such conditions mimic common critical care scenarios, including endotracheal tube obstruction (due to secretions, clots, or plication) and post-extubation airway edema [23, 24]. Our findings showed that Dex exhibited a significant but weak to moderate correlation with ΔPes, ΔPdi, and PTPes. However, both Dex and Tfdi increased significantly under IRL. Additionally, Dex significantly differed between patients who were successfully weaned and those who failed, while Tfdi showed no such distinction.

In this study, the correlations between Dex and indices of breathing effort were weak to moderate, and there was an overlap in values between weaning success and failure, limiting its predictive accuracy. These results can be explained by the fact that; a) ΔPes and ΔPdi are the product of the combined activation of all inspiratory muscles [36] and not only of the diaphragm, b) Dex does not capture isometric contractions of the diaphragm, during which there is no measurable excursion, whereas the pressure–time product of esophageal pressure (PTPes) does account for this [30] and c) under conditions of increased resistive load, the diaphragm may not always be the primary contributor to inspiratory pressure generation, further complicating its relationship with breathing effort [37, 38].

Moreover, Dex is influenced by the interplay between respiratory mechanics, lung volumes, and intrathoracic/intra-abdominal pressures [5, 6, 17, 22]. In addition, the pattern of respiratory muscle activation varies significantly between subjects [36, 39, 40] and leads to variable rib cage and abdominal displacement combinations. Consequently, in Dex there is an increased interpatient variability. Based on these, in spontaneously breathing patients under a resistive load, as our population, Dex may be used as a monitoring tool, assessing temporal trends, but not exact values of breathing effort, and assisting in weaning outcome predictions.

TFdi did not correlate with indices of breathing effort, even though it increased when IRL was applied. Furthermore, it did not differ between patients who succeeded and failed weaning. This does not mean that our study suggests that TFdi is not a helpful index for weaning. There are studies where Tfdi correlated significantly with breathing effort [14, 15, 19, 20] and with weaning outcomes [7–9]. However other studies that align with our results [10, 13, 16–18], showed weak or no correlation between Tfdi, breathing effort and weaning outcomes. Finally, in a previous study [41], TFdi increased during the SBT. While TFdi may have risen even without IRL, the strong temporal association between IRL application and increases in Pes, Pdi and Tfdi may suggest a causal relationship.

Population-related and methodological factors may have contributed to these differences. Prior research has highlighted significant variability in respiratory muscle activation patterns, both in critical care patients [15–17, 21] and in healthy individuals [36–40]. This variability in respiratory muscle recruitment could explain the inconsistent relationship between TFdi and pressure output [15–17]. Specifically, in critical care patients with diaphragmatic dysfunction, an increased activation of extra-diaphragmatic muscles has been observed [15, 21]. Our data may suggest a reduced contribution in respiratory pressures from the diaphragm. This could be attributed to either diaphragmatic dysfunction and/or an increase in extra-diaphragmatic muscles as compensation [15, 21], or to preferential use of the respiratory accessory muscles [36, 39]. In this cohort, depending on the definition used [4, 5], diaphragmatic dysfunction ranged between 50 and 80%, which aligns with the reported rates in the literature [42]. Our population consisted of critical care patients who had been ventilated for longer periods than those in previous studies [7, 14, 15, 19], increasing the likelihood of respiratory muscle dysfunction [43] and possibly weakening the association between TFdi and pressure generation [15]. Unfortunately, the activity of the extra-diaphragmatic muscles was not assessed and as such we cannot provide further insight at this point. Furthermore, different resting lung volumes may have been an additional factor introducing interpatient variability in the diaphragmatic pressure-generating capabilities [44] and should be considered for in future investigations. Other technical issues in respect of the measurement, such as inclusion or not of the pleura, might have also affected the relationships between sonographic indices and breathing effort which were found in the present study [5, 17]. Even though the zone of apposition is considered the place of maximal thickening [45], the force-generating capacity of the diaphragm is not uniform [46] and as such interpatient variances cannot be ruled out [47]. Consequently, the visualized part of the diaphragm might not accurately reflect the function of the whole muscle.

Furthermore, while in almost all successfully weaned patients Tfdi was above the usual cut-offs (> 30–35%), the weaning failure group displayed a wide range of Tfdi values, with the average being above the usual thresholds [4, 7, 9]. This suggests that while TFdi < 30–35% is strongly associated with weaning failure, as previously reported [7], values above this threshold do not necessarily indicate success, potentially reflecting a low negative predictive value in patients with prolonged ventilation and diaphragmatic dysfunction. This aligns with previous studies that showed decreased muscle strength despite an increased Tfdi [48]. Additionally, as there is currently no definitive TFdi cut-off for diagnosing diaphragmatic dysfunction [5], Tfdi may not be used as a universal index for accurately capturing diaphragmatic efficiency. We hypothesize that in these patients, despite having Tfdi values above 30–35%, diaphragmatic inflammatory injury [48] impaired its ability to generate sufficient pressure; the latter may had an important impact in weaning outcome and may have caused a compensatory increase in extra-diaphragmatic muscle activity, as PTPes almost doubled during IRL. As such, in populations such as ours, TFdi may demonstrate greater variability and may not be as reliable in predicting breathing effort, as in other patient groups. Moreover, Tfdi values < 30–35% may be used as a cut-off for weaning failure. Under resistive load, Tfdi, which reflects primarily contractile shortening, may not increase despite higher contractile forces.

Although Dex and TFdi share many similar characteristics [3, 49] (such as the 2D visualization of a 3D structure, limited representation of the respiratory muscle apparatus, and interpatient variability) they differed in their relationship with breathing effort and weaning outcomes. This difference is also reflected in the weak correlation between the two measures [50]. Tfdi assesses diaphragmatic contractility and Dex diaphragmatic displacement and possibly lung volume change [51, 52]. Specifically, at tidal volumes below 50% of inspiratory capacity TFdi reflects active muscle thickening and remains unaffected by lung volume [17]. In patients who rely more on rib cage muscles for inspiration [36, 39], tidal volume may drive diaphragmatic excursion, even with minimal or no increase in TFdi. Our hypothesis is that these characteristics of the two variables, led to the different relationships with breathing effort and weaning outcome.

Previous studies have evaluated breathing mechanics and diaphragm function under IRL [37–40, 53–55]. However, in some of them the breathing pattern was prespecified [38, 53], or the enrolled population was exclusively healthy subjects [37–40, 53, 54] making comparisons difficult. In two studies [53, 54], where diaphragmatic kinetics were assessed via ultrasound during unloaded breathing and IRL, the reported results were results similar to ours, as both Dex and Tfdi increased under IRL. In our study, the Ti/Ttot increased, while the MIF decreased during IRL, aligning with previous findings [37, 38, 54, 55]. However, some differences exist between our results and those of prior studies. For instance, two studies [37, 54] reported an increase in Vt and a decrease in RR, whereas in patients with upper airway obstruction [55], both Vt and RR remained unchanged. In contrast, in patients breathing with an IRL similar to ours [40], Vt decreased.

In our study, Dex correlated significantly with Vt during spontaneous breathing while no significant correlation was found during IRL. Moreover, there was an increase in Dex despite a decrease in Vt during IRL. In the current literature, the strong correlations between Dex and Vt refer mainly on healthy subjects [51, 52] and to our knowledge, there are no data on the relationship between them during resistive breathing in similar populations to ours. However, in a previous study [53], where Vt was kept constant, upon application of the same IRL, Dex increased. Moreover, when bilateral anterolateral magnetic phrenic nerve stimulation is conducted, with the airway occluded Vt does not increase despite the diaphragmatic contraction and displacement [56]. A possible explanation for this uncoupling is that the resistance reduces thoracic amplification. This might seem to counter the argument of increased extra-diaphragmatic muscle activity. However, as shown in previous studies [39, 40] patients breathing against a resistive load exhibited a more elliptical thoracic shape and reduced anteroposterior diameter, as well as lower Vt [40], accompanied by an increased activity of extra-diaphragmatic muscles. Moreover, the resulting thoracic shape appeared to be independent of diaphragmatic activity and was instead influenced by the preferential activation of specific extra-diaphragmatic muscles, such as the parasternals and intercostals. Therefore, we believe that a reduced thoracic expansion might not necessarily indicate decreased extra-diaphragmatic muscle activity; rather, it may reflect an increased contribution of these muscles to the overall inspiratory effort, which aligns with our hypothesis regarding their role in this patient cohort. Another plausible explanation may be that Dex assesses diaphragm at a certain local point which may be dissociated from global lung volume change during the application of resistive load. Measurement of the extra-diaphragmatic muscles activity or, electrical impedance tomography could provide further insight in our findings.

In the present investigation, RSBI in weaning success patients, increased under IRL but it remained within normal limits (< 110 breaths/min/L), unlike in weaning failure patients. It is of note that some patients who failed weaning presented RSBI values below 110 breaths/min/L during spontaneous breathing, but RSBI increased significantly (*p* = 0.016) with the application of IRL. These findings may suggest that patients who are successfully weaned may have greater respiratory reserve, allowing them to tolerate resistive loading without a significant change in their breathing pattern.

To our knowledge, most studies evaluating the relationship between diaphragmatic ultrasound and breathing effort have been conducted in subjects under positive-pressure ventilation, which can confound diaphragmatic ultrasound measurements and alter their relationship with breathing effort [5, 6, 17, 22]. Additionally, diaphragmatic ultrasound exhibits high interpatient variability [49], and its correlation with breathing effort has been inconsistent across studies [10, 14–19]. To address these limitations, we conducted our study during an unassisted spontaneous breathing trial (SBT) to eliminate the confounding effects of positive-pressure ventilation on diaphragm mechanics. To mitigate interpatient variability and focus on the intrapatient relationship between diaphragmatic ultrasound measurements and breathing effort, we employed repeated measures correlation [33], a statistical method designed to assess within-patient associations. Furthermore, the effect of an IRL on breathing mechanics has been studied mainly on healthy subjects. The application of such protocol in critical care patients may be challenging in terms of patient cooperation and clinical stability which are required for valid measurements. Moreover, the idea of applying a restive load during weaning might be perceived by physicians as a challenge which may induce respiratory muscle fatigue. Our rationale was that an IRL for a brief time (1 min) even though it may increase the breathing effort, it may be similar -if not less in magnitude- with a MIP maneuver of 30 s duration and may simulate a clinical scenario of increased resistive load to the respiratory system.

Certain considerations should be taken into account when interpreting our results. The weaning failure rate in this study was high. Similar failure rates have been previously reported [1, 21] while a recent epidemiological study [57], with patients being ventilated for fewer days than in our study, reported a failure rate of up to 35% in first separation attempts. Moreover, we used a composite definition of weaning failure, combining SBT and extubation failure [25]. These two end-points should not be confused as they might have different pathophysiologic basis. Our aim in this study was to assess the relationship between Dex and Tfdi with breathing effort indices in patients undergoing SBT and under resistive load and not to explore specific pathophysiological mechanisms for extubation or SBT outcome. Also, the enrolled population had a longer ventilation duration compared to previous studies [7, 14, 15, 20], it was predominantly composed of males with an average APACHE-II score of 16.2, which is associated with an estimated mortality of 15–30%, and had an increased incidence of diaphragmatic dysfunction, which may have also caused the increased weaning failure rate. These characteristics might limit the generalizability of our findings.

In this study, we elected to use PTPes over PTPdi because PTPes reflects the oxygen cost of the entire respiratory system [30] rather than isolating the diaphragm. Furthermore, the esophageal catheter was calibrated according to the manufacturer’s instructions rather than the previously recommended calibration procedure [58], which may have introduced variability in the measurements. On the other hand, we used a fixed load, rather than one normalized on Pi/Pimax, and this does not account for individual differences in respiratory muscle strength, potentially imposing a higher relative effort on some patients. As such, despite the careful explanation of the maneuvers, fear, anxiety or respiratory muscle fatigue from the increased load may have interfered with our findings and should be considered in a future investigation. Finally, gastric pressure was measured only in thirty-six patients, and one might certainly argue that the ratio of gastric to transdiaphragmatic pressure -and therefore diaphragmatic activity- could have been different in those patients than the rest of our cohort. We certainly cannot exclude that possibility. However, there was no difference between those thirteen patients and the rest of our cohort in terms of parameters associated with diaphragmatic dysfunction such as the duration of ventilation, cause of admission, Dex, Tfdi, ΔPes or demographics (data not shown).

In conclusion, in patients with prolonged mechanical ventilation breathing through a t-piece and under resistive load, Dex had a weak to moderate relationship with indices of breathing effort and thus may not be used to accurately predict them. Dex differed significantly between weaning outcomes but there was an overlap of values between the two groups. TFdi did not correlate significantly with breathing effort, under conditions of increased resistive load in this study. Future studies which could evaluate the interplay between the diaphragm and extra-diaphragmatic muscles under similar conditions might provide further insight in the relationship between diaphragmatic sonography and breathing effort.

## Data Availability

The data that support the findings of this study are available from the corresponding author, upon reasonable request.
